# The impact of the UK COVID-19 pandemic on patient-reported health outcomes after stroke: a retrospective sequential comparison

**DOI:** 10.1007/s00415-021-10819-9

**Published:** 2021-10-15

**Authors:** Hatice Ozkan, Gareth Ambler, Gargi Banerjee, Edgar Chan, Simone Browning, John Mitchell, Richard Perry, Alex P. Leff, Robert J. Simister, David J. Werring, the SIGNaL collaborators, Rolf Jager, Nick Ward, Parashkev Nachev

**Affiliations:** 1 Stroke Research Centre, Queen Square Institute of Neurology, University College London, London, UK; 2 Comprehensive Stroke Service, National Hospital for Neurology and Neurosurgery, Institute of Neurology, University College London Hospitals NHS Foundation Trust, Queen Square, London, WC1N UK; 3 Department of Statistical Science, University College London, Gower Street, London, UK; 4 MRC Prion Unit at UCL, Institute of Prion Diseases, University College London, London, UK; 5 Neuropsychology Department, National Hospital for Neurology and Neurosurgery Queen Square, London, WC1N 3BG UK

**Keywords:** Stroke, Patient-reported health outcomes, COVID-19, Ischaemic stroke, Intracerebral haemorrhage

## Abstract

**Background and purpose:**

The COVID-19 pandemic and related social isolation measures are likely to have adverse consequences on community healthcare provision and outcome after acute illnesses treated in hospital, including stroke. We aimed to evaluate the impact of the COVID-19 pandemic on patient-reported health outcomes after hospital admission for acute stroke.

**Methods:**

This retrospective study included adults with acute stroke admitted to the University College Hospital NHS Foundation Trust Hyperacute Stroke Unit. We included two separate cohorts of consecutively enrolled patients from the same geographical population at two time points: 16th March–16th May 2018 (pre-COVID-19 pandemic); and 16th March–16th May 2020 (during the COVID-19 pandemic). Patients in both cohorts completed the validated Patient Reported Outcomes Measurement Information System–29 (PROMIS-29 version 2.0) at 30 days after stroke.

**Results:**

We included 205 patients who were alive at 30 days (106 admitted before and 99 admitted during the COVID-19 pandemic), of whom 201/205 (98%) provided patient-reported health outcomes. After adjustment for confounding factors, admission with acute stroke during the COVID-19 pandemic was independently associated with increased anxiety (*β* = 28.0, *p* < 0.001), fatigue (*β* = 9.3, *p* < 0.001), depression (*β* = 4.5, *p* = 0.002), sleep disturbance (*β* = 2.3, *p* = 0.018), pain interference (*β* = 10.8, *p* < 0.001); and reduced physical function (*β* = 5.2, *p* < 0.001) and participation in social roles and activities (*β* = 6.9, *p* < 0.001).

**Conclusion:**

Compared with the pre-pandemic cohort, patients admitted with acute stroke during the first wave of the COVID-19 pandemic reported poorer health outcomes at 30 day follow-up in all domains. Stroke service planning for any future pandemic should include measures to mitigate this major adverse impact on patient health.

**Supplementary Information:**

The online version contains supplementary material available at 10.1007/s00415-021-10819-9.

## Introduction

The ongoing global COVID-19 pandemic caused by the severe acute respiratory syndrome coronavirus 2 (SARs-CoV-2), first documented in Wuhan, China [1], has spread to more than 200 countries and resulted in over 4.6 million deaths worldwide [2]. Many countries introduced major public health measures in an attempt to slow down community spread of the virus. In the UK, the primary strategy was a national “lockdown” with enforced widespread social isolation and shielding of vulnerable people (https://www.gov.uk/government/speeches/pm-statement-on-coronavirus-16-march-2020) [3]. These measures included: closure of educational institutions, workplaces and places of worship; bans on public events and international travel; movement restrictions; limiting physical interactions (including visiting family and friends); working from home where possible; one form of exercise a day; and only contacting doctors or general practitioners via telephone or video call^3^. Overnight these measures transformed societal behaviour, inter-community links, and social and clinical care services; this has led to concerns about delays in clinical treatments and social support with increased regional disparities in health outcomes [4].

People with pre-existing or acute medical conditions may be more vulnerable to the psychosocial effects of a pandemic and associated public health measures [5]. In particular, health outcomes after acute stroke may be severely impacted by pandemic restrictions associated with fewer stroke admissions (especially for those with less severe strokes concerned about catching COVID-19) [6], restricted family or carer contact in hospital, and reduced support networks including community rehabilitation [7–9]. A recent meta-analysis found that depressed mood, anxiety, impaired memory, and sleep disturbance were present in 33–42% of patients admitted to hospital for severe acute respiratory syndrome or Middle East respiratory syndrome, and that in some cases these effects were prolonged [10]. Furthermore, social deprivation and isolation are associated with unfavourable psychological outcomes, functional dependency and premature mortality among stroke survivors [11].

We are not aware of systematic research on the adverse impact of the first wave of the UK COVID-19 pandemic on patient-reported health outcomes following acute stroke. The aim of this study was to evaluate the impact of the pandemic on patient-reported health outcome domains by comparing a cohort of patients admitted during the pandemic to a pre-pandemic cohort drawn from the same geographical population, with adjustment for potential confounding factors.

## Methods

### Study design, setting and population

We reviewed prospectively collected data in patients presenting with stroke to the Hyperacute Stroke Unit (HASU) at University College Hospital (UCH) which provides stroke care to an ethnically diverse population of approximately 1.4 million people in North Central London. Since February 2015, routine clinical data from all patients have been included in an ongoing registry study, Stroke Investigation in North and Central London (SIGNaL). We included patients who presented during two time periods: pre-pandemic (16th March–16th May 2018) and during the first wave of the pandemic (16th March 2020–16th May 2020).

**Fig. 1:**
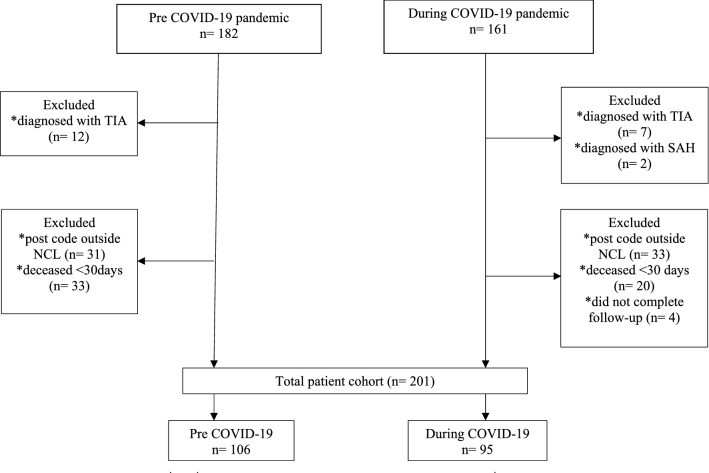
Flow chart of patients included pre- and during the first wave of the COVID-19 pandemic. *COVID-19* coronavirus disease-2019, *TIA* transient ischaemic attack, *SAH* subarachnoid haemorrhage, *NCL* North Central London

Patients were included if they were: aged > 18 years; resident in the North Central London boroughs of Enfield, Haringey, Barnet, Highbury and Islington, or Camden; diagnosed with ischaemic stroke (IS) or intracerebral haemorrhage (ICH) confirmed on brain imaging; and had completed at least two domains of the patient-reported health outcome scale (PROMIS-29; Patient-Reported Outcomes Measurement Information System) at 30-day follow-up (see Fig. 1). During both the pre-pandemic and pandemic periods, patients were followed up at 30 days post stroke as part of standard care.

### Sociodemographic and clinical characteristics

The clinical and sociodemographic variables of interest (i.e., those that are important to describe the included population or likely to affect patient-reported health outcomes) were identified prior to the study by two senior authors (DJW and RJS). These included demographic characteristics (age, sex, ethnic origin, and discharge location), medical history, stroke type (ischaemic stroke or intracerebral haemorrhage), admission stroke severity (assessed by the National Institutes of Health Stroke Scale (NIHSS) score), cardiovascular risk factors, medication at hospital discharge, dementia diagnosis, disability at hospital discharge (measured with the modified Rankin Scale (mRS)), and whether the patient had any general practitioner contact after hospital discharge.

### Health outcome measurement

The primary outcome, PROMIS-29, consists of seven patient-reported health outcome domains that capture physical function, anxiety, depression, fatigue, sleep disturbance, participation in social roles and activities, pain interference (with work, social life, household tasks and daily activities) and pain intensity (using a visual analogue scale). Each domain contains four items and is assessed on a 5-point Likert response with values ranging from 1 to 5 except for the sub-domain on pain intensity, which is scored on 11-point numeric rating scale (0–10). The raw domain scores are converted into T scores and standardised to the US general population (mean, 50: SD = 10) [12]; the pain intensity sub score is averaged and determined as 0 being no pain to 10 being the worst imaginable pain [13]. The formula for calculation of PROMIS-29 domain scores can be found at: (https://www.healthmeasures.net/score-and-interpret/calculate-scores). In our analysis, we have oriented all of the PROMIS-29 domain scores so that higher mean scores always indicate worse patient-reported health outcomes.

### Standard protocol approvals and patient consents

The SIGNaL registry of routinely collected clinical data is approved by the University College Hospitals NHS Foundation Trust Governance Review Board as a continuous service evaluation of a comprehensive clinical care programme (service evaluation 5-201920-SE); for this reason, informed patient consent was not required.

### Statistical analysis

After data were entered, cleaned and verified, we compared data between pre-versus during the COVID-19 pandemic group using descriptive statistics. Continuous variables were compared using either the unpaired *t* test or Wilcoxon rank sum test, and categorical variables were compared using chi squared test or Fisher’s exact test, as appropriate. Categorical variables are presented as percentages, and continuous variables as mean (standard deviation (SD)) or median (inter-quartile range (IQR)). Missing baseline data were handled using multiple imputation by chained equations [14, 15] using ethnicity, discharge destination, cardiovascular risk factors and length of hospital stay to create 18 complete datasets.

Univariable analysis using independent t tests was used to test for changes and differences in each health outcome domain between the groups (pre- versus during the COVID-19 pandemic). We checked normality using the Jarque–Bera goodness-of-fit test, and where appropriate, nonparametric distributional diagnostic plots and visual inspection of the histograms and quantile normal plots were used. For the multivariable adjusted linear regression model, we included variables that were judged as potentially relevant a priori, reached the pre-defined statistical significance level of *p* ≤ 0.20 in univariable analyses, or both (sex, age, stroke type, dementia, heart disease, previous stroke/TIA, admission NIHSS, length of HASU stay, discharge mRS, discharge destination, ethnicity, antihypertensive, smoking status, general practitioner visits and time to follow-up). Variance inflation factors (VIFs) were used to check for multicollinearity.

Multivariable linear regression models were also constructed to determine other predictors (in addition to pre- or during pandemic status) for each patient-reported domain. Covariates were determined either a priori for clinical relevance or by an alpha significance level of < 0.20 in univariable analyses (see Supplementary Table 1). We did not adjust for COVID-19 diagnosis as the number was very small (*n* = 18) and as this was a feature of the pandemic, we did not regard it as a confounding factor. Backwards elimination at alpha level of < 0.10 was used to identify all additional predictors (apart from pre- or during pandemic status) associated with each health domain score. All statistical analysis was carried out by Hatice Ozkan (MSc) and Gareth Ambler (Ph.D.), University College London.

## Results

### Patient characteristics

The flow chart for patient inclusion is shown in Fig. 1. Of the 258 stroke patients that met the inclusion criteria, 205 patients were alive at 30-day follow-up; of those, 201 (pre-COVID-19 = 106, during the COVID-19 pandemic = 95) patients (98%) completed the PROMIS-29 outcome measure. Table 1 summarises the clinical and sociodemographic characteristics of patients admitted pre- and during the COVID-19 pandemic. There were no significant differences in age (mean 71.0 vs 70.4 years), sex (females 61.3% vs 55%), stroke type (ischaemic stroke or intracerebral haemorrhage) or the proportions of patients receiving intravenous thrombolysis or thrombectomy) between the two groups. However, compared with the pre-pandemic cohort, the cohort studied during the pandemic had: more severe strokes (median NIHSS score 6 vs 4.5); a higher proportion of patients from Black or Asian ethnic groups (24.2% vs 12.3% and 26.3% vs 11.3%, respectively); more severe disability at hospital discharge (median mRS 3 vs 2); a longer HASU stay (4 vs 3 days); a higher proportion of patients receiving early supported discharge (53.7% vs 34%); and a higher proportion of patients who did not see a general practitioner after discharge (41% vs 19.1%). Patients seen before the pandemic more often had a history of heart disease (21% vs 10.5%) and more frequent antiplatelet drug use (67.0% vs 52.6%). We identified significant difference in time to follow-up between the groups (pre-COVID-19 34 days versus during COVID-19 = 32 days); the proportion of proxy responders (next of kin or carer) was lower during the pandemic (20.7% vs 28.9%).

**Table 1:** Patient characteristics pre- and during the COVID-19 pandemic

Variable	*N*	Pre- COVID-19 pandemic	During the COVID-19 pandemic	*p* value
		106	95	
Female sex (%)	*n* (%)	65 (61.3%)	52 (55%)	0.345^†^
Intravenous tPA	*n* (%)	15 (14.2%)	12 (12.6%)	0.708
Thrombectomy	*n* (%)	4 (3.8%)	2 (2.1%)	0.306
Age (years)	Mean (SD)	71.0 ± 14.2	70.4 ± 16.5	0.8058*
Stroke type				
Ischaemic stroke	*n* (%)	91 (85.8%)	81 (85.2%)	0.906^†^
ICH	*n* (%)	15 (14.2%)	14 (15.0%)	–
Ethnicity				
White	*n* (%)	60 (56.6%)	40 (42.1%)	< 0.001^†^
Asian	*n* (%)	12 (11.3%)	25 (26.3%)	–
Black	*n* (%)	13 (12.3%)	23 (24.2%)	–
Other	*n* (%)	21 (20%)	7 (7.3%)	–
Medical history and risk factors				
Hypertension	*n* (%)	73 (68.9%)	70 (73.7%)	0.452^†^
Diabetes mellitus	*n* (%)	24 (22.6%)	29 (30.5%)	0.205^†^
Atrial fibrillation	*n* (%)	29 (27.4%)	20 (21.1%)	0.299^†^
Previous stroke/TIA	*n* (%)	27 (25.5%)	20 (21.1%)	0.460^†^
Hypercholesterolemia	*n* (%)	37 (34.9%)	35 (36.8%)	0.775^†^
Heart disease	*n* (%)	22 (21.0%)	10 (10.5%)	0.048^†^
Dementia	*n* (%)	12 (11.3%)	9 (9.5%)	0.669^†^
Smoking history	*n* (%)	40 (37.7%)	27 (28.4%)	0.162^†^
COVID-19 positive	*n* (%)	0	18 (19.0%)	–
Current medication on hospital admission				
Anticoagulants	*n* (%)	22 (20.8%)	21 (22.1%)	0.816^†^
Antiplatelet	*n* (%)	71 (67.0%)	50 (52.6%)	0.038^†^
Antihypertensive	*n* (%)	52 (49.1%)	59 (62.1%)	0.063^†^
Statin	*n* (%)	80 (75.5%)	73 (76.8%)	0.820^†^
Baseline severity measures, discharge destination and follow-up				
Discharge mRS	Median (IQR)	2 (1–3)	3 (1–5)	0.0094*
30-day mRS	Median (IQR)	1 (0–2)	3 (1–4)	0.0324*
NIHSS on admission	Median (IQR)	4.5 (2 –7)	6 (3–12)	0.0213*
Length of HASU stay (days)	Median (IQR)	3 (2–4)	4 (2–7)	0.0527*
Discharge location				
Home with ESD	*n* (%)	36 (34.0%)	51 (53.7%)	0.018^‡^
ASU/care home	*n* (%)	54 (51%)	35 (36.8%)	–
Time to follow-up, days	Median (IQR)	34 (30–40)	32 (30–34)	0.0029*
Proxy responders	*n* (%)	30 (28.9%)	19 (20.7%)	0.186^†^
Not seen GP after discharge	*n* (%)	20 (19.1)	38 (41%)	0.001^†^

ICH, intracerebral haemorrhage; tPA, tissue plasminogen activator; TIA, transient ischaemic attack; COVID-19, corona virus disease- 2019; mRS, modified Rankin Scale; NIHSS, NIH stroke scale; HASU, hyperacute stroke unit; ESD, early supported discharge; ASU, acute stroke unit; G* p*, general practitioner

Values are *n* (%) or median (IQR)

Numbers that do not add up to the appropriate totals or percentages that do not add up to 100% are a result of missing data

^*^Mann–Whitney *U* test comparing  pre- vs during COVID-19

^†^χ^2^ test

^‡^Fisher’s exact test

### Patient-reported health outcomes

**Fig. 2:**
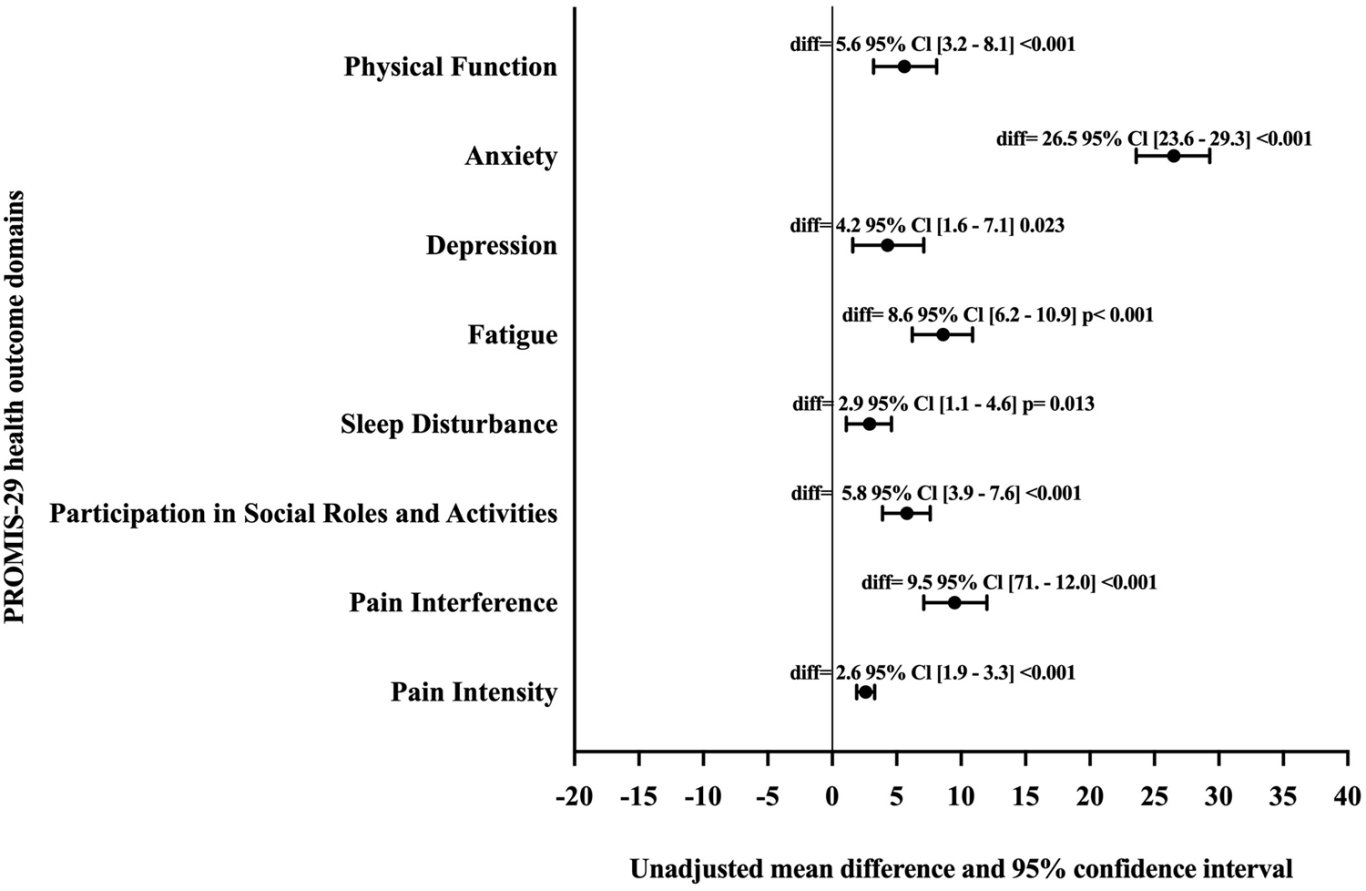
Point estimates to the right indicate worse health outcomes for physical function, anxiety, depression, fatigue, sleep disturbance, participation in social roles and activities pain interference, and pain intensity. Bold circles show between-group mean change and error bars show 95% confidence intervals of each domain

Unadjusted group changes and between group differences are reported in Table 2 and Fig. 2. Compared to the pre-COVID-19 pandemic group, the mean score for all domains of PROMIS-29 indicated worse outcomes during the COVID-19 pandemic, with significantly higher anxiety (mean difference = 26.5, 95% Cl 23.6–29.3 *p* < 0.001), depression (mean difference = 4.2, 95% Cl 1.6–7.1 *p* = 0.023), fatigue (mean difference = 8.6, 95% Cl 6.2–10.9 *p* < 0.001), and pain interference (mean difference = 9.5, 95% Cl 7.1–12.0 *p* < 0.001). There were lower scores for physical function (mean difference = 5.6, 95% Cl 3.2–8.1 *p* < 0.001) and participation in social roles and activities (mean difference = 5.8, 95% Cl 3.9–7.6 *p* < 0.001). Percentage of patients with scores meaningfully worse than the pre-COVID-19 pandemic ranged from 43.8% for physical function to 74.7% in anxiety (see Table 2).

**Table 2:** Patient-reported health domain scores in stroke patients admitted pre- versus during the COVID-19 pandemic

Health outcome domain	Pre-COVID-19 pandemic (*n* = 106), mean ± SD [95% Cl]	During the COVID-19 pandemic (*n* = 95), mean ± SD [95% Cl]	*p* value
Physical function	55.8 ± 9.7 [54.1–57.6]	61.4 ± 7.4 [60.1–62.8]	< 0.001
Anxiety	38.1 ± 11.7 [35.8–40.3]	64.6 ± 8.5 [60.6–66.2]	< 0.001
Depression	53.5 ± 10.7 [51.4–55.5]	57.8 ± 9.0 [56.0–59.7]	0.0130
Fatigue	55.5 ± 9.4 [53.7–57.3]	64.0 ± 7.1 [62.7–65.6]	< 0.001
Sleep disturbance	54.7 ± 6.0 [53.5–55.8]	57.6 ± 6.6 [56.3–59.0]	0.0009
Participation in social roles and activities	54.0 ± 6.2 [52.8–55.2]	59.8 ± 7.0 [58.4–61.2]	0.001
Pain interference	51.2 ± 8.4 [49.6–52.8]	60.7 ± 9.2 [59.0–62.7]	0.001
Pain intensity (0–10)	2.6 ± 2.3 [2.2–3.1]	5.2 ± 2.6 [4.8–5.8]	0.001

Each domain mean score (except for pain intensity) has a range from 20 to 80; a sore of > 50 indicates meaningfully worse health than the general population. Pain intensity is rated from 0 to 10 on a visual analogue score

**Fig. 3:**
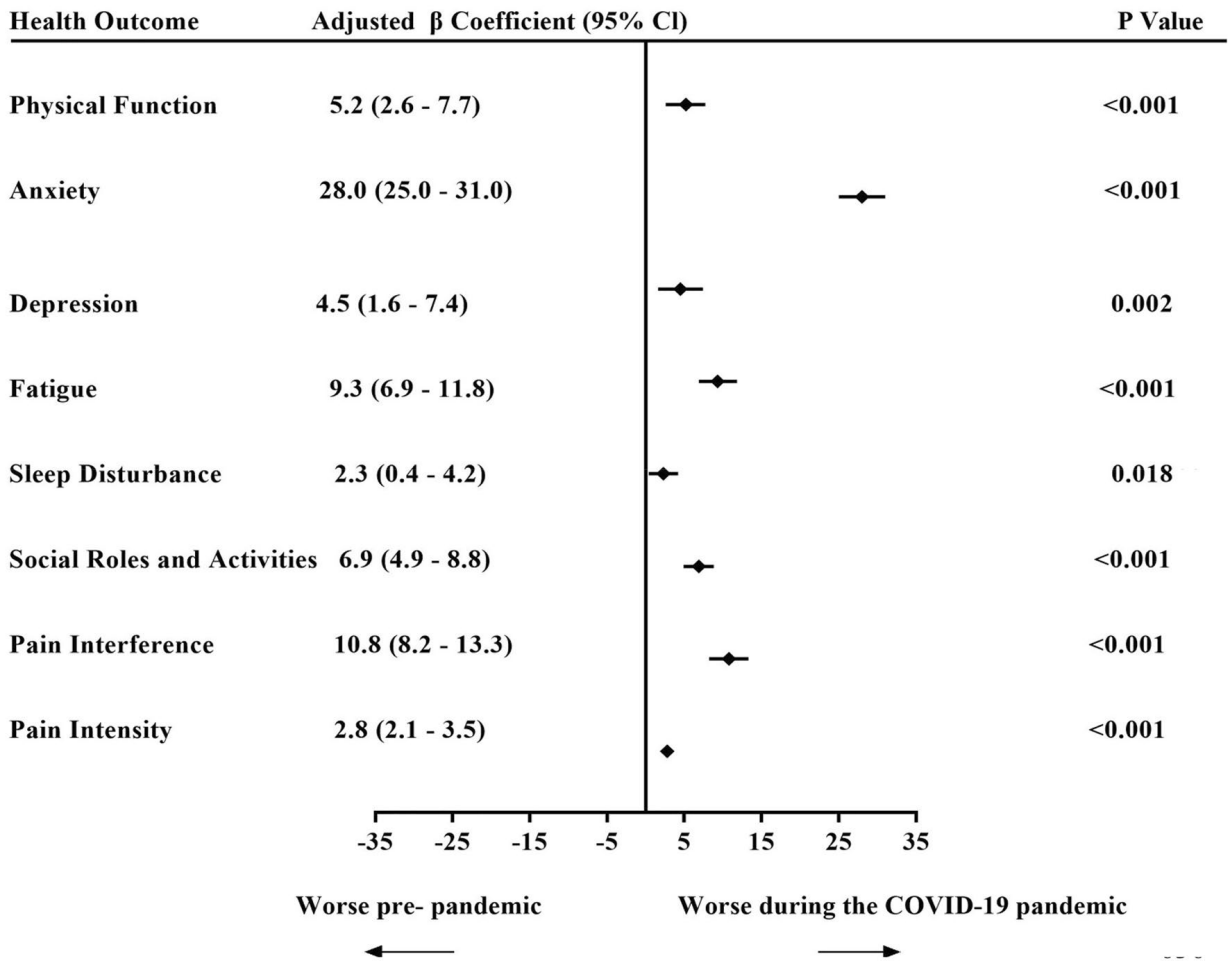
Adjusted beta coefficients from multivariable linear regression showing associations of the COVID-19 pandemic with patient-reported health domains. Multivariable linear regression model showing the association between admission to stroke unit during COVID-19 pandemic and the adjusted *β* coefficients (with 95% confidence intervals and *p* values) from separate multivariable linear regression models for each patient-reported health outcome score, adjusted for potential confounders and variables selected by *p* < 0.2 in univariable analyses: (age, sex, stroke type, dementia, heart disease, previous stroke/TIA, admission NIHSS, length of stroke unit stay, discharge mRS, discharge destination, ethnicity, antihypertensive, smoking status, general practitioner visit, proxy responder and time to follow-up)

After adjusting for clinically relevant and other potentially confounding covariates determined in univariable analysis (Table 1), admission during the COVID-19 pandemic was independently associated with worse patient-reported health for all PROMIS domains in multivariate regression models, including anxiety *β* = 28.0 95% Cl [25.0–31.0] *p* < 0.001; fatigue *β* = 9.3 95% Cl [6.9–11.8] *p* < 0.001; and pain interference *β* = 10.8 95% Cl [8.2–13.3] *p* < 0.001 (see Fig. 3). The association between other covariates and patient-reported health varied by domain. However, admission pre-COVID-19 pandemic, and discharge to home with early supported (ESD) was associated with better reported health in most domains. By contrast,  admission during the pandemic, moderate to severe disability at hospital discharge (mRS 3–5), black ethnic origin, history of heart disease, no ESD support and not seeing a general practitioner after discharge were associated with worse health in multiple domains.

## Discussion

Our data clearly show that stroke survivors treated during the COVID-19 pandemic reported substantially worse patient-reported health outcomes at 30 day follow-up, even after adjusting for potential confounding factors. Compared to the pre-pandemic cohort, patients admitted during the pandemic had worse health outcomes in all domains, including increased anxiety, depression, fatigue, sleep disturbance, and pain, with reduced physical function and social participation. Although all domains were affected, we found the largest differences in patient-reported anxiety (which almost doubled), pain interference, fatigue and social participation. Since stroke is the commonest cause of adult complex disability in the UK, the magnitude and consistency of these worsened health outcomes suggest that the COVID-19 pandemic has led to major unmet healthcare needs for stroke survivors, with immediate clinical relevance for acute and community stroke care pathways.

Possible explanations for significantly worse patient-reported health outcomes during the pandemic include behavioural factors and direct effects of the pandemic such as: decrease in community care; a lack of informal social support; reduced monitoring of severe symptoms in the community; lack of direct links to primary care; increased loneliness; the challenges of living in confined households; limited availability of remote healthcare interventions; and increased anxiety about the trajectory of stroke recovery [16].

We are not aware of other systematic studies of patient-reported health outcomes after acute stroke during COVID-19 lockdowns, although such measures are considered essential for managing, responding, and planning recovery from a pandemic [17]. However, our findings might not be specific to patients with acute stroke. Previous studies also reported significant associations between COVID-19-related restrictions and adverse mental health and quality of life outcomes in other groups, including the general population, healthcare workers, children, older people, and patients with cancer or Parkinson’s disease [16, 18–24]. Most previous studies were restricted to the general population [1, 6–13, 16–20], frontline healthcare staff [16], the elderly [21] or children [22]. Unfortunately, we do not have sufficiently detailed data from previous studies to assess whether the magnitude of the impact of COVID-19 changes is greater after stroke than other conditions. This question could be addressed in future studies.

Although a recent online UK survey of 1546 stroke survivors from Stroke Association (2020) also investigated patient-reported health outcomes associated with COVID-19, only 5.49% of respondents had a stroke during the pandemic. By contrast with our study, this survey had a limited response rate, did not include a cohort representative of the overall stroke population, had no control group, and did not use a validated standardised health outcome instrument. Nevertheless, the high reported rates of anxiety and depression, with reduced access to post-stroke support are consistent with our findings, which confirm and build on these observations. Moreover, two other small studies also reported adverse psychological outcomes associated with COVID-19, albeit with significant methodological limitations. Ahmed et al. [25] reported significant increase in post-stroke anxiety and depression related to social deprivation, but the study did not include a comparator group, was limited to small study cohort (*n* = 52) and included a large proportion of patients of Arab origin. Pisano et al. [26] also found increased anxiety in aphasic stroke survivors during the pandemic, but this study cohort was not representative of the full stroke population. By contrast, we included a well-phenotyped and ethnically diverse population of stroke survivors with a control group drawn from the same geographical population.

Although previous studies show that post-stroke pain affects around 40% of stroke survivors [27] we are not aware of studies of post-stroke pain during the COVID-19 pandemic. We identified that during the pandemic pain interference and intensity was significantly higher compared those seen pre-pandemic. One explanation could be lack of access to social networks, limited social support, reduction in access to general practitioners during the pandemic, inability to access face-face rehabilitation or access prescribed opioids or other painkillers [28]. In our secondary analysis (supplementary document, Table 1), we identified stroke severity, lack of a general practitioner visits and Black ethnic origin as significant predictors of a worse pain interference score.

We found markedly worse fatigue and sleep disturbance during the pandemic first wave (compared to the pre-pandemic period) consistent with observations that the phrase “pandemic fatigue” resulted in around 200 million Google search engine hits, and searches for “pandemic insomnia” increased by 58% [29, 30]. Although data on post-stroke fatigue during the COVID-19 pandemic remain very limited, our findings are consistent with two recent studies including the Stroke Association [31] online survey and a small observational cohort study (*n* = 28) from [32] which had methodological limitations. The increase in fatigue during the pandemic in comparison to pre-pandemic levels may reflect altered perceptions of fatigue during a global health crisis, or its coexistence with other health comes such as anxiety, depression and sleep disturbance. Our findings of worse reported sleep during the pandemic are also consistent with reports in the general population and healthcare workers during the COVID-19 pandemic [33–36]; but only a very small proportion of stroke survivors were included in these studies. Possible contributing factors to post-stroke sleep disturbance include lack of physical activity, increased anxiety, depression, social isolation, post-traumatic stress, and reduced in-person [37].

The finding of reduced social participation after stroke during the first wave COVID-19 lockdown was expected, since social isolation and shielding were the primary public health strategies to reduce viral spread, impacting on many social aspects of life [38, 39]. Worryingly, social isolation has adverse effects on rehabilitation compliance, engagement in activities of daily living, anxiety and premature death amongst stroke patients [40–43]. However, data on how mandatory isolation affects stroke survivor’s social participation are extremely limited [7, 25]. Potential adverse consequences of decreased social participation include an inability to work with remote technology, lack of face-face contact with social networks, reduction in family roles such as taking care of grand-children, lack of access to daycentres and places of worship [3].

During the pandemic, we identified significantly worse physical functioning compared to the pre-pandemic period. This finding could be related to reduced access to rehabilitation which could only be accessed via virtual (online) routes, which were not widely available. This may also be challenging for stroke survivors who may have limited access and skills for the use of the required technology, especially if they have cognitive or language impairments [44]. However, Chen et al. [45] and Raefsky et al. [46] found no difference in physical function of stroke survivors who received remote rehabilitation versus those receiving standard care, despite patients in the remote group spending 10% more time with therapists and being younger. In line with our observations, Cieza et al. [47] reported rushed hospital discharges, absence of routine follow-up, moderate to severe functional disability at hospital discharge, lower back pain and limited face-to-face healthcare as significant predictors of decline in physical function, though these findings were not specific to stroke only and included limited information on patient characteristics.

Our study has important strengths. We investigated the impact of the first wave of the COVID-19 pandemic on key patient-reported health outcomes in an ethnically diverse representative North London stroke population. We collected follow-up data in 98% of eligible patients using a validated instrument (PROMIS-29). We avoided dichotomisation of patient-reported health outcomes to retain statistical power and eliminate loss of descriptive quantitative information in the study population [48]. We included a pre-pandemic control population from the same geographical region and detailed phenotype data allowed us to adjust for confounding factors, including those related to the altered spectrum of stroke characteristics during the pandemic.

Limitations include the relatively small sample size from a single centre and a control population from 2018, so there may have been a change in healthcare trends that we were not able to fully adjust for (for example changes in hospital medical care or general practitioner behaviour). Although many previous studies in stroke have investigated longer-term outcomes, we chose to focus on 30-day outcome data to identify the early and direct impact of healthcare changes during the pandemic, including rapid discharge from hospital, less face-face rehabilitation, early follow-up, and community care. Future studies should investigate the impact of the COVID-19 pandemic on longer-term outcomes after acute stroke. In summary, compared to patients admitted with acute stroke during a pre-pandemic period, patients seen during the COVID-19 pandemic had worse patient-reported health outcomes including neuropsychological, physical and social participation domains. The magnitude and consistency of these worsened health outcomes suggest that the COVID-19 pandemic has led to a major unmet healthcare need for stroke survivors, with immediate clinical relevance for acute and community stroke care pathways.

## Supplementary Information

Below is the link to the electronic supplementary material.

Supplementary file1 (DOCX 23 KB)

## References

[1] Zhou F, Yu T, Du R (2020). Clinical course and risk factors for mortality of adult inpatients with COVID-19 in Wuhan, China: a retrospective cohort study. Lancet.

[2] Coronavirus disease (COVID-19)—World Health Organization. https://www.who.int/emergencies/diseases/novel-coronavirus-2019. (Accessed 17 Sept 2021)

[3] Prime Minister’s statement on coronavirus (COVID-19): 16 March 2020. GOV.UK. https://www.gov.uk/government/speeches/pm-statement-on-coronavirus-16-march-2020. (Accessed 24 Mar 2021)

[4] Paremoer L, Nandi S, Serag H, Baum F (2021). COVID-19 pandemic and the social determinants of health. BMJ.

[5] Pfefferbaum B, North CS (2020). Mental health and the COVID-19 pandemic. N Engl J Med.

[6] Perry RJ, Smith CJ, Roffe C (2020). Characteristics and outcomes of COVID-19 associated stroke: a UK multicentre case–control study. J Neurol Neurosurg Psychiatry.

[7] Liu L, Wang D, Brainin M, Elkind MSV, Leira E, Wang Y (2020). Approaches to global stroke care during the COVID-19 pandemic. Stroke Vasc Neurol.

[8] Nguyen-Huynh MN, Tang XN, Vinson DR (2020). Acute stroke presentation, care, and outcomes in community hospitals in Northern California during the COVID-19 pandemic. Stroke.

[9] Carolin H, Anne E, Huttner HB (2020). Acute stroke in times of the COVID-19 pandemic. Stroke.

[10] Rogers JP, Chesney E, Oliver D (2020). Psychiatric and neuropsychiatric presentations associated with severe coronavirus infections: a systematic review and meta-analysis with comparison to the COVID-19 pandemic. Lancet Psychiatry.

[11] Bray BD, Paley L, Hoffman A (2018). Socioeconomic disparities in first stroke incidence, quality of care, and survival: a nationwide registry-based cohort study of 44 million adults in England. Lancet Public Health.

[12] Deyo RA, Ramsey K, Buckley DI (2016). Performance of a Patient Reported Outcomes Measurement Information System (PROMIS) short form in older adults with chronic musculoskeletal pain. Pain Med.

[13] Katzan IL, Lapin B (2018). PROMIS GH (Patient-Reported Outcomes Measurement Information System Global Health) scale in stroke. Stroke.

[14] Royston P (2004). Multiple imputation of missing values. Stata J.

[15] Aloisio KM (2014). Analysis of partially observed clustered data using generalized estimating equations and multiple imputation. Stata.

[16] Chudasama YV, Gillies CL, Zaccardi F (2020). Impact of COVID-19 on routine care for chronic diseases: a global survey of views from healthcare professionals. Diabetes Metab Syndr.

[17] Aiyegbusi OL, Calvert MJ (2020). Patient-reported outcomes: central to the management of COVID-19. Lancet.

[18] Wang Y, Shi L, Que J (2021). The impact of quarantine on mental health status among general population in China during the COVID-19 pandemic. Mol Psychiatry.

[19] Bu F, Steptoe A, Fancourt D (2020). Loneliness during strict lockdown: trajectories and predictors during the COVID-19 pandemic in 38,217 adults in the UK. medRxiv.

[20] Clair R, Gordon M, Kroon M, Reilly C (2021). The effects of social isolation on well-being and life satisfaction during pandemic. Human Social Sci Commun.

[21] Lam K, Lu AD, Shi Y, Covinsky KE (2020). Assessing telemedicine unreadiness among older adults in the united states during the COVID-19 pandemic. JAMA Intern Med.

[22] Newlove-Delgado T, McManus S, Sadler K (2021). Child mental health in England before and during the COVID-19 lockdown. Lancet Psychiatry.

[23] Bargon CA, Batenburg MCT, van Stam LE, van der Molen DRM, van Dam IE, van der Leij F (2021). Impact of the COVID-19 pandemic on patient-reported outcomes of breast cancer patients and survivors. JNCI Cancer Spectrum.

[24] Guo D, Han B, Lu Y, Lv C, Fang X, Zhang Z (2020). Influence of the COVID-19 pandemic on quality of life of patients with Parkinson’s disease. Parkinson’s Disease.

[25] Ahmed ZM, Khalil MF, Kohail AM, Eldesouky IF, Elkady A, Shuaib A (2020). The prevalence and predictors of post-stroke depression and anxiety during COVID-19 pandemic. J Stroke Cerebrovasc Diseases.

[26] Pisano F, Giachero A, Rugiero C, Calati M, Marangolo P (2020). Does COVID-19 impact less on post-stroke aphasia? This is not the case. Front Psychol.

[27] Payton H, Soundy A (2020). The experience of post-stroke pain and the impact on quality of life: an integrative review. Behav Sci (Basel).

[28] Blanca F, Alonso LDM, Sebastián GM (2021). Stroke acute management and outcomes during the COVID-19 outbreak. Stroke.

[29] Zitting K-M, der Holst HML, Yuan RK, Wang W, Quan SF, Duffy JF (2021). Google Trends reveals increases in internet searches for insomnia during the 2019 coronavirus disease (COVID-19) global pandemic. J Clin Sleep Med.

[30] The concept of “fatigue” in tackling COVID-19—the BMJ. https://blogs.bmj.com/bmj/2020/10/26/the-concept-of-fatigue-in-tackling-covid-19/. (Accessed 24 Mar 2021) 10.1136/bmj.m4171 33139254

[31] Stroke Association, 2021. Stroke recoveries at risk report. [online] Stroke Association. https://www.stroke.org.uk/stroke-recoveries-at-risk-report. (Accessed 10 Jan 2021)

[32] Chuan Q, Luoqi Z, Ziwei H (2020). Clinical characteristics and outcomes of COVID-19 patients with a history of stroke in Wuhan, China. Stroke.

[33] Casagrande M, Favieri F, Tambelli R, Forte G (2020). The enemy who sealed the world: effects quarantine due to the COVID-19 on sleep quality, anxiety, and psychological distress in the Italian population. Sleep Med.

[34] Polenick CA, Daniel NR, Perbix EA (2021). Factors associated with sleep disturbances related to the COVID-19 pandemic among older adults with chronic conditions. Am J Geriatr Psychiatry.

[35] Christiansen J, Lund R, Qualter P, Andersen CM, Pedersen SS, Lasgaard M (2021). Loneliness, social isolation, and chronic disease outcomes. Ann Behav Med.

[36] Huang Y, Zhao N (2020). Generalized anxiety disorder, depressive symptoms and sleep quality during COVID-19 outbreak in China: a web-based cross-sectional survey. Psychiatry Res.

[37] Hinkle JL, Becker KJ, Kim JS (2017). Poststroke fatigue: emerging evidence and approaches to management: a scientific statement for healthcare professionals from the American Heart Association. Stroke.

[38] Hwang T-J, Rabheru K, Peisah C, Reichman W, Ikeda M (2020). Loneliness and social isolation during the COVID-19 pandemic. Int Psychogeriatr.

[39] Wong A, Ho S, Olusanya O, Antonini MV, Lyness D (2020). The use of social media and online communications in times of pandemic COVID-19. J Intensive Care Soc.

[40] Vincent-Onabajo GO (2013). Social participation after stroke: one-year follow-up of stroke survivors in Nigeria. ISRN Stroke.

[41] Thorne K, Williams JG, Akbari A, Roberts SE (2015). The impact of social deprivation on mortality following acute myocardial infarction, stroke or subarachnoid haemorrhage: a record linkage study. BMC Cardiovasc Disord.

[42] Bejot Y, Daubail B, Giroud M (2016). Epidemiology of stroke and transient ischemic attacks: current knowledge and perspectives. Revue Neurologique.

[43] Harrison AS, Gaskins NJ, Connell LA, Doherty P (2020). Factors influencing the uptake of cardiac rehabilitation by cardiac patients with a comorbidity of stroke. IJC Heart Vasc.

[44] Mantovani E, Zucchella C, Bottiroli S (2020). Telemedicine and virtual reality for cognitive rehabilitation: a roadmap for the COVID-19 pandemic. Front Neurol.

[45] Chen T, Zhang B, Deng Y, Fan J-C, Zhang L, Song F (2019). Long-term unmet needs after stroke: systematic review of evidence from survey studies. BMJ Open.

[46] Raefsky S, Dodakian L, Le V et al (2020) Social factors related to home-based telerehabilitation after stroke (1431). Neurology 94(15 Supplement). https://n.neurology.org/content/94/15_Supplement/1431. (Accessed 3 Mar 2021)

[47] Cieza A, Causey K, Kamenov K, Hanson SW, Chatterji S, Vos T (2020). Global estimates of the need for rehabilitation based on the Global Burden of Disease study 2019: a systematic analysis for the Global Burden of Disease Study 2019. Lancet.

[48] Cumberland PM, Czanner G, Bunce C (2014). Ophthalmic statistics note: the perils of dichotomising continuous variables. Br J Ophthalmol.

