# Supplementary material for: The impact of the UK COVID-19 pandemic on patient-reported health outcomes after stroke: a retrospective sequential comparison
Source: J Neurol. 2021 Oct 15;269(4):1741–50. doi: 10.1007/s00415-021-10819-9 (PMC8517937; doi:10.1007/s00415-021-10819-9)
Supplement: Supplementary file 1 — Supplementary file1 (DOCX 23 KB) [file 415_2021_10819_MOESM1_ESM.docx]

**The impact of the first wave of the UK COVID-19 pandemic on patient-reported health outcomes after stroke: retrospective sequential comparison**

**Supplemental material**

| Table 1 Secondary Multivariable linear regression models predicting domain scores in patients admitted pre versus during the COVID-19 pandemic | | | | | | | | | | | | | | | |
| --- | --- | --- | --- | --- | --- | --- | --- | --- | --- | --- | --- | --- | --- | --- | --- |
|  | Physical Function | | Anxiety | | Depression | | Fatigue | | Sleep Disturbance | | Social roles and activities | Pain Interference | | Pain Intensity | |
| COVID-19 Pandemic | 5.2 (1.3) ^a, b^ | | 28.0 (1.5) ^a, b^ | | 4.5 (1.4) ^a, b^ | | 9.3 (1.2) ^a, b^ | | 2.3 (1.0) ^a, b^ | | 6.9 (1.8) ^a, b^ | 10.8 (1.3) ^a, b^ | | 2.8 (0.4) ^a, b^ | |
| Age (per year) | -0.07 (0.0) ^b^ | | -0.1 (0.7) | | 0.1 (1.7) | | -0.1 (1.4) | | -1.3 (1.1) | | 0.1 (1.1) | 0.04 (0.1) | | 1.3 (0.5) ^b^ | |
| Female sex | 1.0 (1.0) | | 0.5 (1.9) | | 0.3 (1.7) | | -2.4 (1.2) ^b^ | | 2.6 (1.7) ^a, b^ | | 1.0 (1.7) | 2.3 (1.4) ^a, b^ | | 1.6 (1.2) ^b^ | |
| Stroke type (reference: ischaemic stroke) | | | | | | | | | | | | | | | |
| ICH | -0.4 (1.5) | | 3.7 (2.6) ^a, b^ | | 3.6 (2.3) ^a, b^ | | 1.8 (2.0) ^b^ | | 0.2 (1.4) | | -3.2 (1.2) ^b^ | 2.3 (2.0) | | 0.1 (0.2) | |
| Ethnicity (reference: white) | | | | | | | | | | | | | | | |
| Asian | 3.6 (1.6) ^a, b^ | | -3.6 (2.5)^a, b^ | | 3.4 (2.1) | | -1.5 (1.9) | | 1.9 (1.4) ^b^ | | -2.4 (1.7) ^b^ | -0.8 (1.9) | | 0.4 (0.5) | |
| Black | 1.4 (1.4) | | -0.7 (2.5) | | 0.1 (2.2) | | 3.6 (1.8) ^a, b^ | | 0.1 (1.4) | | -0.3 (2.1) | 4.7 (2.0) ^a, b^ | | 0.02 (0.5) | |
| Other | 1.3 (1.5) | | 2.4 (2.7) | | 3.4 (2.4) | | -0.7 (1.9) | | 0.2 (1.5) | | -0.2 (2.3) | -2.2 (2.1) | | -0.3 (0.6) | |
| Medical history | | | | | | | | | | | | | | | |
| Previous stroke/TIA | -0.02 (1.2) | | 0.9 (2.1) | | 3.1 (2.3) ^b^ | | 1.3 (1.6) | | -0.04 (1.2) | | 2.9 (1.9) ^b^ | -0.1 (1.6) | | 0.6 (0.5) | |
| Dementia | 1.7 (1.6) | | 3.8 (2.9) | | -0.8 (2.2) | | 1.3 (2.2) | | 2.2 (0.5) ^b^ | | 0.8 (2.5) | -0.6 (2.2) | | -0.5 (0.6) | |
| Heart disease | 1.2 (1.4) | | 5.6 (2.5) ^a, b^ | | -0.4 (2.2) | | 2.9 (1.8) ^b^ | | 1.2 (1.3) | | 2.6 (1.2) | 1.5 (1.9) | | 0.5 (0.4) | |
| Smoking | 3.3 (1.3) ^a, b^ | | -0.5 (2.1) | | 2.4 (1.8) | | -2.2 (1.6) | | -0.4 (1.1) | | 0.5 (1.7) | -0.1 (1.6) | | -0.2 (0.3) | |
| Baseline measures | | | | | | | | | | | | | | | |
| Admission NIHSS | -0.02 (0.1) | | -0.1 (0.2) | | 0.02 (0.4) | | -0.5 (0.1) | | -0.2 (0.1) | | 0.2 (0.4) | 0.2 (0.1) ^a, b^ | | 0.7 (0.4) | |
| Discharge mRS | 4.8 (1.7) ^a, b^ | | -1.3 (0.7) | | 0.7 (0.6) | | 1.0 (0.5) | | -0.2 (0.4) | | 3.2 (2.1) ^a, b^ | 4.5 (1.8) ^a, b^ | | -0.4 (0.1) | |
| Length of stroke unit stay | -0.2 (0.1) | | 0.1 (0.2) | | 0.1 (0.2) | | 0.7 (0.2) | | 1.2 (0.7) ^b^ | | 0.5 (0.2) ^a, b^ | -0.2 (0.2) | | -0.1 (0.2) | |
| Discharge destination (reference: home, no ESD) | | | | | | | | | | | | | | | |
| Home ESD | -1.0 (1.6) | -4.7 (1.7) ^a, b^ | | -2.4 (2.5) ^b^ | | -2.3 (1.4) ^b^ | | -1.6 (1.4) | | 0.1 (2.4) | | | -4.1 (1.3) ^a, b^ | | -1.7 (0.6) ^b^ |
| ASU/Care Home | 1.2 (1.1) | -0.6 (1.9) | | -1.1 (1.7) | | -0.7 (1.5) | | 4.1 (1.1) ^a, b^ | | 2.5 (1.7) ^b^ | | | -0.6 (1.5) | | 0.2 (0.4) |
| Time to follow-up | -0.9 (1.3) | -0.2 (0.2) | | -0.1 (0.2) | | 0.02 (1.7) | | -0.1 (0.2) | | -0.1 (0.2) | | | -0.1 (0.5) | | -0.01 (0.1) |
| Proxy response | 1.2 (1.3) | 2.9 (2.8) | | 1.0 (1.9) | | 5.2 (1.7) ^a, b^ | | 1.6 (1.2) | | 0.5 (1.8) | | | 2.2 (1.7) | | 0.6 (0.5) |
| Not seen GP | -0.2 (1.2) | 4.0 (1.7) ^a, b^ | | 0.6 (1.9) | | 2.4 (1.7) ^a, b^ | | -0.9 (1.2) | | -0.7 (0.2) | | | 3.0 (1.5) ^b^ | | 2.3 (1.7) ^a, b^ |
| Abbreviations: COVID-19= Corona virus disease-2019; ICH= Intracerebral haemorrhage; TIA= Transient ischaemic attack; NIHSS= NIH stroke scale; mRS= modified Rankin Scale; ASU= Acute stroke unit; GP= General practitioner. Data shown are β coefficients (standard error (SE)) from separate multivariable linear regression models for each domain using the same covariates. ^a^ p <0.001; ^b^ p<0.05; ^a, b^ variable meaningfully associated with worse (higher >50) domain score. | | | | | | | | | | | | | | | |
